# Reassessing Banana Phylogeny and Organelle Inheritance Modes Using Genome Skimming Data

**DOI:** 10.3389/fpls.2021.713216

**Published:** 2021-08-11

**Authors:** Chung-Shien Wu, Edi Sudianto, Hui-Lung Chiu, Chih-Ping Chao, Shu-Miaw Chaw

**Affiliations:** ^1^Biodiversity Research Center, Academia Sinica, Taipei, Taiwan; ^2^Plant Germplasm Division, Taiwan Agricultural Research Institute, Taichung, Taiwan; ^3^Taiwan Banana Research Institute, Pingtung, Taiwan

**Keywords:** banana, genome skimming, cytoplasmic inheritance, nrDNA, mitogenome, plastome

## Abstract

Bananas (*Musa* spp.) are some of the most important fruit crops in the world, contributing up to US$10 billion in export values annually. In this study, we use high-throughput sequencing to obtain genomic resources of high-copy DNA molecules in bananas. We sampled 13 wild species and eight cultivars that represent the three genera (*Ensete, Musa*, and *Musella*) of the banana family (Musaceae). Their plastomic, 45S rDNA, and mitochondrial scaffolds were recovered from genome skimming data. Two major clades (Clades I & II) within *Musa* are strongly supported by the three genomic compartment data. We document, for the first time, that the plastomes of Musaceae have expanded inverted repeats (IR) after they diverged from their two close relatives, Heliconiaceae (the lobster-claws) and Strelitziaceae (the traveler's bananas). The presence/absence of *rps19* within IR regions reinforces the two intra-generic clades within *Musa*. Our comparisons of the bananas' plastomic and mitochondrial DNA sequence trees aid in identifying hybrid bananas' parentage. As the mitochondrial genes of *Musa* have elevated substitution rates, paternal inheritance likely plays an influential role on the *Musa* mitogenome evolution. We propose genome skimming as a useful method for reliable genealogy tracing and phylogenetics in bananas.

## Introduction

Musaceae, the banana family, comprises ca. 91 species in three genera: *Ensete, Musella*, and *Musa* (Christenhusz and Byng, 2016). Bananas are one of the major fruit crops in many developing countries (Padam et al., 2014). It is estimated that >100 million tons of bananas are produced annually since 2009, with the export value of US$13.5 billion in 2019 (Food Agriculture Organization of the United Nations, 2021). Despite their enormous socio-economic value, cultivated bananas are threatened by various diseases and pests, such as *Fusarium* wilt (Panama disease), black leaf streak, and nematode infection, and by frequent drought due to climate change (Bakry et al., 2009; Brown et al., 2020). Genetic improvements through hybridization have thus been the preferred way to obtain resistant cultivars that ensure sustainable and safe banana production (Ortiz, 2013).

*Musa* L., the largest banana genus, contains four sections: *Australimusa*, 2*n* = 2*x* = 20; *Callimusa*, 2*x* = 18 or 20; *Eumusa*, 2*x* = 22; *Rhodochlamys*, 2*x* = 22 (Cheesman, 1947). However, this traditional classification has been revised with *Eumusa* and *Rhodochlamys* merged into the sect. *Musa* (or Clade I), and *Australimusa* and *Callimusa* into the sect. *Callimusa* (Clade II) based on numerous molecular studies (e.g., Li et al., 2010; Christelová et al., 2011; Janssens et al., 2016; Lamare et al., 2017). Many edible bananas arose from intra- or inter-specific hybridizations between *Musa acuminata* (*Eumusa*; A genome) and *M. balbisiana* (*Eumusa*; B genome), resulting in various cultivars with AA, BB, AB, AAA, AAB, ABB, AAAA, AAAB, AABB, and ABBB genomes (Simmonds and Shepherd, 1995). In addition, *M. schizocarpa* (*Eumusa*; S genome) and *M. textilis* (*Australimusa*; T genome) have contributed to the genetic pool in banana breeding programs (Pereira and Maraschin, 2015; Christelová et al., 2017).

Numerous molecular techniques have been developed to differentiate banana cultivars, including restriction fragment length polymorphism (RFLP; Gawel et al., 1992), cytogenetics (Osuji et al., 1997; CíŽková et al., 2015), microsatellites (SSR; Mattos et al., 2010; Christelová et al., 2017), diversity arrays technology markers (DArT; Sardos et al., 2016), and DNA barcodes (Hribová et al., 2011; Dhivya et al., 2020). However, the complex breeding history of bananas may further complicate efforts to identify accessions and trace their origins. For example, extensive studies showed frequent occurrences of chromosomal recombination (e.g., reciprocal translocation or chromosomal inversion) in bananas (Baurens et al., 2018; Martin et al., 2020a,b; Cenci et al., 2021). These genome structural diversities have been applied to study the origins of banana cultivars and *Musa* evolution (Martin et al., 2020b).

Fauré et al. (1994) firstly reported the differential inheritance modes of organellar genomes in bananas; the plastid genomes (plastomes) are maternally inherited, while the mitochondria (mitogenomes) are passed down from fathers. This unusual cytoplasmic inheritance mode facilitates the tracing of hybrid banana origins (Carreel et al., 2002; Boonruangrod et al., 2008). However, previous studies relied on complex molecular marker patterns to estimate genealogies. We therefore propose an alternative method, genome skimming, to retrieve high-copy DNA sequences (e.g., nuclear ribosomal DNA (rDNA), plastomes, and mitogenomes; Straub et al., 2012; Dodsworth, 2015) to determine parentage in bananas. Genome skimming enables generation of sequence assemblies from high-copy DNA molecules without the need for primer design, PCR amplification, electrophoretic assays, cloning, and Sanger sequencing. In the era of next generation sequencing (NGS), this approach is more reliable and less tedious than genotyping or RFLP analyses but has not yet been applied in estimating banana genealogies or phylogenies.

To verify if our proposed method can be used in lineage and hybrid identification, we used an artificial interspecific hybrid, *M* × *formobisiana*, as one of our study materials. This hybrid cultivar originated from a controlled cross between a maternal parent *M. itinerans* var. *formosana* and a paternal parent *M. balbisiana* (Chiu et al., 2017). We assembled the plastomes, 45S rDNAs, and mitochondrial gene sequences of 21 banana accessions using the genome skimming approach. We further show that these sequences are useful to study the banana phylogeny.

Moreover, the distinct inheritance modes between plastomes and mitogenomes of bananas provide an excellent opportunity to compare mutational loads in paternal and maternal gametes, as proposed by Greiner et al. (2015). Mitogenome mutation rates of land plants are known to be the slowest among the three genetic compartments (Wolfe et al., 1987; Drouin et al., 2008). In this study, we examine whether this still holds in bananas.

## Materials and Methods

### Material Collection, DNA Extraction, Sequencing, and Assembly

We sampled 21 accessions from live collections of the Taiwan Banana Research Institute and the Taiwan Agricultural Research Institute. These taxa were identified by experts and represent all three genera of the banana family (Table 1). For each sample, approximately 2 g of young leaves were harvested for DNA extraction using a modified CTAB method that includes 0.1% of polyvinylpyrrolidone (PVP-40, Sigma) in the extraction buffer (Stewart and Via, 1993). DNA libraries were constructed using Ovation Rapid Library Preparation kits (NuGEN) and sequenced on an Illumina HiSeq 4000 platform in Tri-I Biotech Company (New Taipei City, Taiwan) to generate pair-end reads of 2 × 150 bp. After quality trimming using Trimmomatic 0.38 (Bolger et al., 2014) with the parameters of leading = 3, trailing = 3, slidingwindow = 4:15, and minlen = 50, these reads were *de-novo* assembled using SPAdes 3.14.0 (Bankevich et al., 2012) with kmer lengths = 21, 33, 55, 77, and 91. We used NOVOPlasty 4.3.1 (Dierckxsens et al., 2017) and GetOrganelle 1.7.4 (Jin et al., 2020) to obtain complete plastome sequences with the reference from the plastid scaffolds generated by SPAdes. Base-scale corrections were conducted using Pilon 1.24 (Walker et al., 2014).

**Table 1:** List of the 21 *Musaceae* species sequenced in this study.

**Section**	**Species**	**Subspecies/Subgroup**	**Common name**	**Collection^**a**^**	**MGIS^**b**^ accession number**	**Country of origin**	**Nuclear genotype**
	*Ensete glaucum*	–	–	TBRI	–	–	–
	*Musella lasiocarpa*	–	–	TBRI	–	–	–
*Australimusa*	*Musa textilis*	–	–	TARI	–	Philippines	TT
*Callimusa*	*Musa beccarii*	–	–	TBRI	V01961	–	–
	*Musa coccinea*	–	–	TBRI	–	–	–
*Eumusa*	*Musa acuminata*	Cavendish	Pei Chiao	TBRI	V0085	Taiwan	AAA
	*Musa acuminata*	Cavendish	Formosana	TBRI	–	Taiwan	AAA
	*Musa acuminata*	Red	Morado	TBRI	V0079	Taiwan	AAA
	*Musa acuminata*	Cavendish	Williams Hybrid	TBRI	V0071	Australia	AAA
	*Musa acuminata*	Pisang lilin	Pisang Lilin	TBRI	V0010	Malaysia	AA
	*Musa acuminata*	Sucrier	Kluai Khai	TBRI	V0190	Thailand	AA
	*Musa acuminata*	*macrocarpa*	–	TARI	–	–	AA
	*Musa acuminata*	z*ebrina*	–	TBRI	V0199	Thailand	AA
	*Musa × chiliocarpa*	–	Boyang	TBRI	V0089	Taiwan	AAB
	*Musa balbisiana*	–	–	TBRI	V0013	Honduras	BB
	*Musa itinerans var. formosana*	–	–	TBRI	–	Taiwan	–
	*Musa × formobisiana*	–	–	TARI	–	Taiwan	–
*Rhodochlamys*	*Musa laterita*	–	–	TBRI	V0197	–	–
	*Musa ornata*	–	–	TBRI	V0198	–	–
	*Musa velutina*	–	–	TBRI	V0177	Australia	–
	*Musa siamensis*	–	–	TARI	–	–	–

a *Specimens from Taiwan Banana Research Institute (TBRI) or Taiwan Agricultural Research Institute (TARI)*.

b *MGIS: Musa Germplasm Information System*.

### Sequence Identification and Annotation

Using BLAST+ 2.10.0 (Camacho et al., 2009) with a maximum E-value of 1E^−10^, 45S rDNA, plastid, and mitochondrial scaffolds were searched against an in-house database that includes publicly available 45S rDNA, plastid, and mitochondrial sequences of monocots. Protein-coding genes on the scaffolds were annotated using the “Transfer Annotation” function in Geneious 11.1.5 (https://www.geneious.com), followed by manual adjustments.

### Sequence Alignment and Phylogenetic Tree Construction

Protein-coding and rDNA sequences were aligned using MUSCLE (Edgar, 2004) implemented in MEGA 7.0.26 (Kumar et al., 2016) with “Codons” and “DNA” options, respectively. These alignments were concatenated to generate specific data matrices for plastid, 45S rDNA, and mitochondrial sequences. We used IQ-TREE 1.5.12 (Nguyen et al., 2015) for partition analysis and model testing and then inferred maximum-likelihood (ML) trees with the standard non-parametric bootstrap of 1,000 replicates. The resulting trees were condensed using a 50% majority-rule consensus and visualized in MEGA.

### Nucleotide Substitution Rates and Statistic Test

Pairwise nucleotide substitution rates between *Musa* accessions were estimated in MEGA with the substitution model = Kimura 2-parameter model, rates among sites = uniform rates, and data treatment = pairwise deletion. Paired sample *t*-tests were performed with the Bonferroni correction in R 3.6.2 (https://www.R-project.org/).

## Results

### Copy-Number Differentiation Among Plastid, 45S, and Mitochondrial DNAs

**Figure 1:**
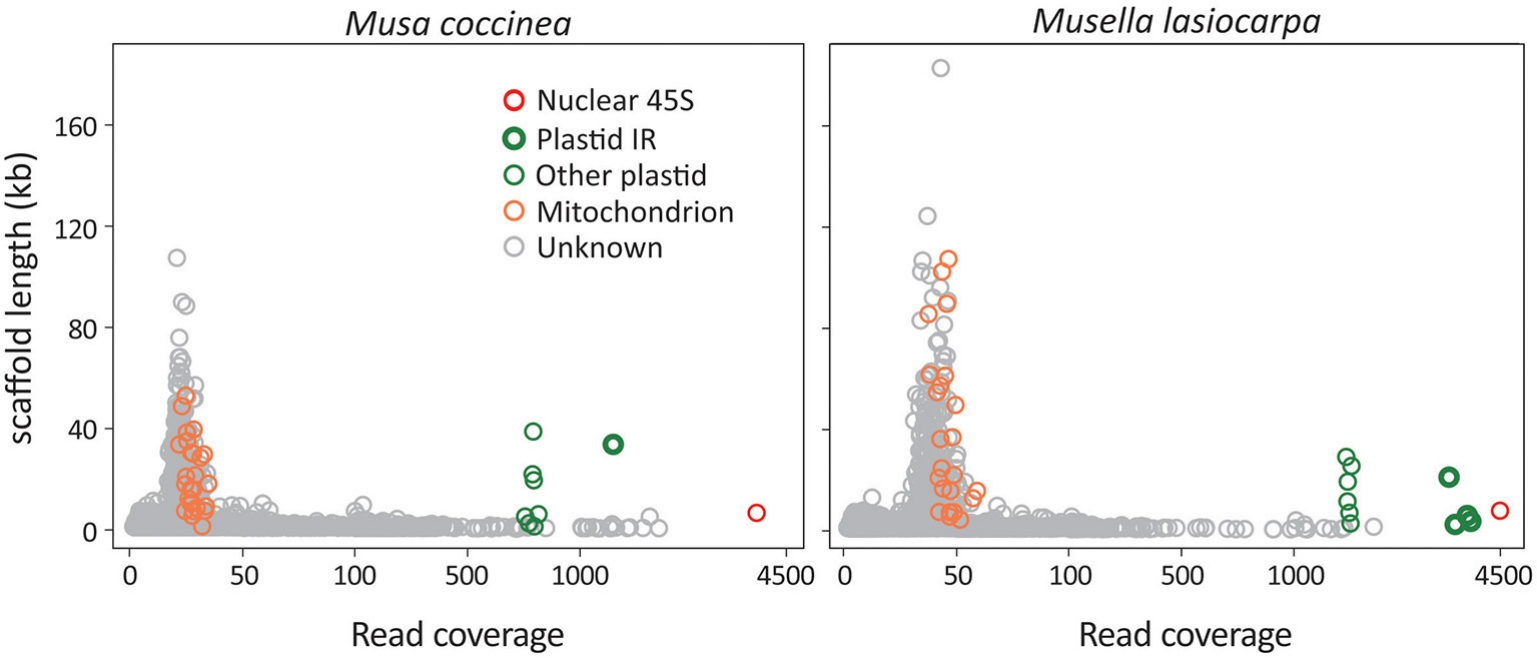
Scatter plots of scaffold lengths vs. read coverage. *Musa coccinea* and *Musella lasiocarpa* are used as the representatives. Red, green, and orange circles indicate 45S rDNA, plastid, and mitochondrial scaffolds, while gray are “unknown” scaffolds that could not be identified using BLAST searches against our in-house database. Some plastid scaffolds are identified as IR regions because their read coverage is approximately double of the other plastid scaffolds.

We generated a total of 1.9–2.3 Gb of sequence consisting of 12.7–15.7 million reads for the 21 sampled taxa (Supplementary Table 1). After *de novo* assembly, we removed scaffolds that significantly deviated from the mean read coverage because they were likely DNA fragments of intracellular horizontal transfers, such as nuclear organelle DNA (norgs) and mitochondrial plastid DNA (mtpts). Among the retained scaffolds, the 45S rDNA scaffold has the highest read coverage, followed by plastid and mitochondrial scaffolds (Figure 1). The read coverage differences are due to copy number variation among scaffolds in the three subgenomes.

Plastome scaffolds fall into two separate groups in our analyses (Figure 1). One of the scaffolds has approximately twice as many reads as the other, indicating that the former is the inverted repeat (IR) sequence, while the latter represents the single-copy (SC) region. These plastid scaffolds were subsequently used as the initial seed for successive rounds of read extension, eventually yielding circular plastomes ranging from 167,700 to 171,982 bp in length (Supplementary Figure 1). In summary, we obtained a single scaffold for both plastomes and 45S rDNAs and 17–32 scaffolds for mitogenomes of each sequenced taxon (Supplementary Table 1). All sequences utilized in this study and their GenBank accession numbers are listed in Supplementary Table 2.

### Plastid Phylogenomics of Musaceae

**Figure 2:**
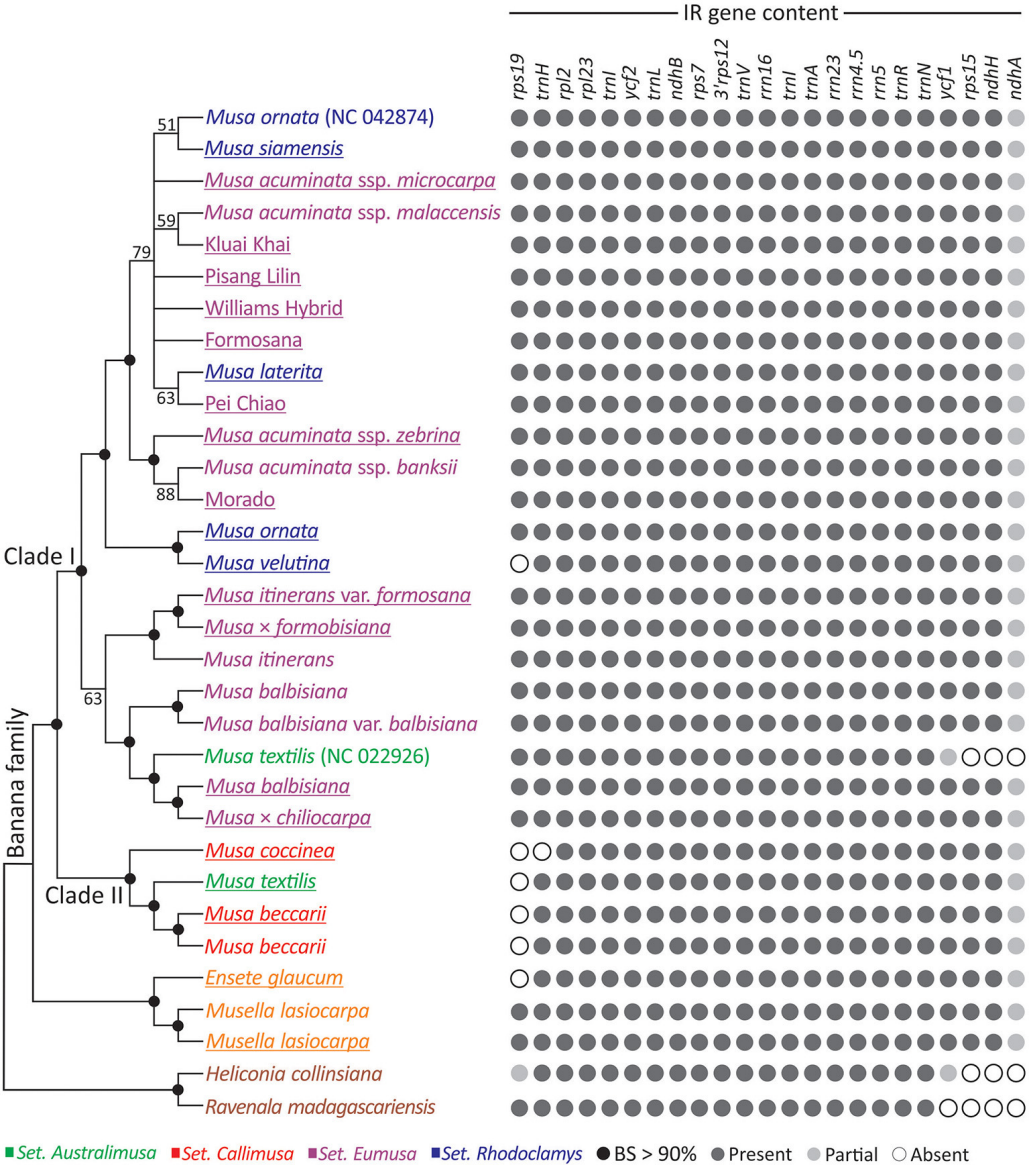
Plastid phylogeny and IR gene content of banana species. An ML tree inferred from the concatenation of 83 plastid genes is shown in the **left** panel, while the IR gene content of the associated taxa is illustrated in the **right** panel. The underlined taxa are sequenced in this study. BS, bootstrap support.

We inferred an ML tree for the three banana genera based on the concatenated plastid protein-coding genes (Figure 2). Figure 2 shows that *Ensete* and *Musella* form a monophyletic group sister to *Musa*. Within *Musa*, two clades (Clades I and II), are strongly supported, each containing taxa from several sections. Clade I includes taxa from three sections: *Australimusa, Eumusa*, and *Rhodoclamys*, while Clade II includes those from two sections: *Australimusa* and *Callimusa*. None of the four sections appears to be monophyletic in this ML tree. Moreover, we found that *M. textilis, M. ornata*, and *M. acuminata* are paraphyletic. *M. ornata* (NC_042874), *M. siamensis*, and *M. laterita* are nested within the accessions of *M. acuminata* (Figure 2). The publicly available *M. textilis* plastome sequence (NC_022926) and our sequenced *M. textilis* are placed separately in Clade I and II. Similarly, our newly sequenced *M. ornata* is sister to *M. velutina* rather than to its conspecific accession NC_042874. To clarify if this species-level paraphyly was due to taxonomic misidentification, we constructed a *matK*-based tree with additional sampling (Supplementary Figure 2). In the *matK* tree, our *M. textilis* and *M. ornata* accessions are each clustered together with at least two conspecific bananas, while no accession is sister to either NC_022926 or NC_042874 (Supplementary Figure 2). These data strongly suggest that NC_022926 and NC_042874 might have been mistakenly labeled in previous studies. By removing both problematic sequences, Clade I contains only *Eumusa* and *Rhodoclamys*, in good agreement with several previous molecular studies (Li et al., 2010; Christelová et al., 2011; Janssens et al., 2016; Lamare et al., 2017).

### The Presence of *rps19* in the IR Distinguishes the Two *Musa* Clades

To explore the IR dynamics and its implication across the banana family, patterns of IR gene content were plotted next to the ML tree (Figure 2). We found that the IR of *M. textilis* (NC_022926) lacks *ycf1, rps15*, and *ndhA*, but these genes are retained in the IRs of other bananas, including our sequenced *M. textilis*. We excluded NC_022926 from our IR comparison because of the uncertainty in its identity discussed above. Martin et al. (2013) reported that an IR expansion has resulted in duplication of *ycf1, rps15, ndhH*, and partial *ndhA* in *Musa*. Using both *Ensete* and *Musella* plastomes as the outgroup, we found that the IR expansion seems to be a synapomorphic trait that distinguishes the banana family from its two close relatives, Heliconiaceae (represented by *Heliconia*) and Strelitziaceae (represented by *Ravenala*). Within *Musa*, Clade I is separated from Clade II by the presence of *rps19* in IRs, except for *M. velutina*, which has a species-specific IR reduction (Figure 2). These structural differences reinforce distinctive intra-generic clades within *Musa*.

### Nuclear 45S rDNAs as Promising Super-Barcodes

**Figure 3:**
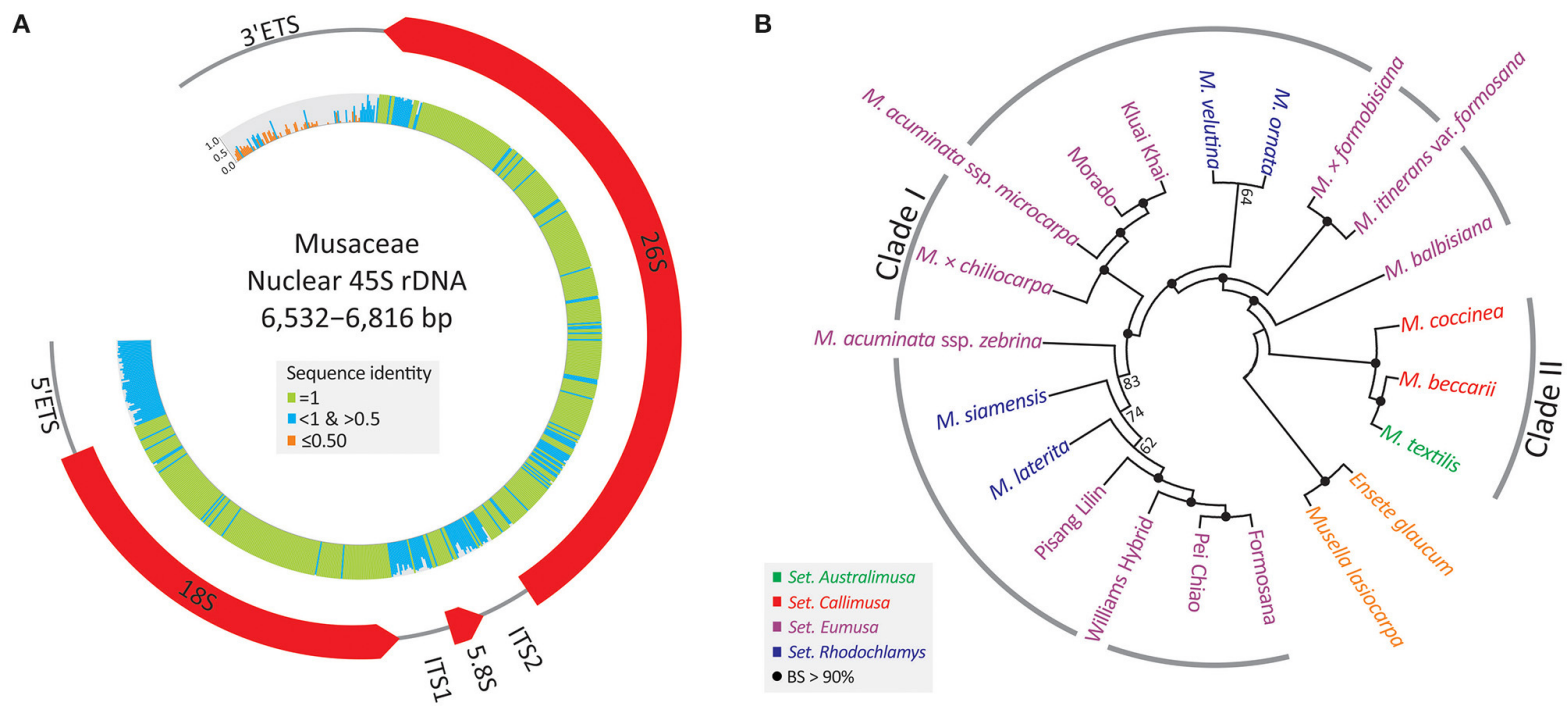
Assemblies of 45S rDNAs in the banana family. **(A)** A map depicting the banana 45S rDNA, including 5'ETS, *18S*, ITS1, *5.8S*, ITS2, *26S*, and 3'ETS. Sequence identity across the 45S rDNA is shown in the inner histograms. **(B)** An unrooted ML tree inferred from the entire 45S rDNA sequence. BS, bootstrap support.

A single 45S rDNA scaffold containing an array of 5'ETS, *18S*, ITS1, *5.8S*, ITS2, *26S*, and 3'ETS (Figure 3A) was recovered from each sampled individual. These nrDNA sequences range from 6,532 to 6,816 bp. They exhibit high sequence divergence at four loci, (3'ETS, 5'ETS, ITS1, and ITS2), where identical sites are nearly absent.

Figure 3B depicts an unrooted ML tree inferred from all 45S rDNA sequences. The major discrepancy between 45S rDNA and plastid trees is the relative position of a triploid (AAB) banana, *M*. × *chiliocarpa*. This banana is clustered with *M. balbisiana* in the plastid tree (Figure 2), but it is nested within the accessions of *M. acuminata* in the 45S rDNA tree (Figure 3B). Nonetheless, the two trees are congruent in support of the two intra-generic clades (i.e., Clades I and II) within *Musa*. Moreover, all taxa, including cultivars, are fully resolved using a 50% majority consensus rule in the 45S rDNA tree (Figure 3B), suggesting that 45S rDNA is an effective super-barcode for discriminating banana species and cultivars.

### Mitochondrial Gene Repertoire of Musaceae

**Figure 4:**
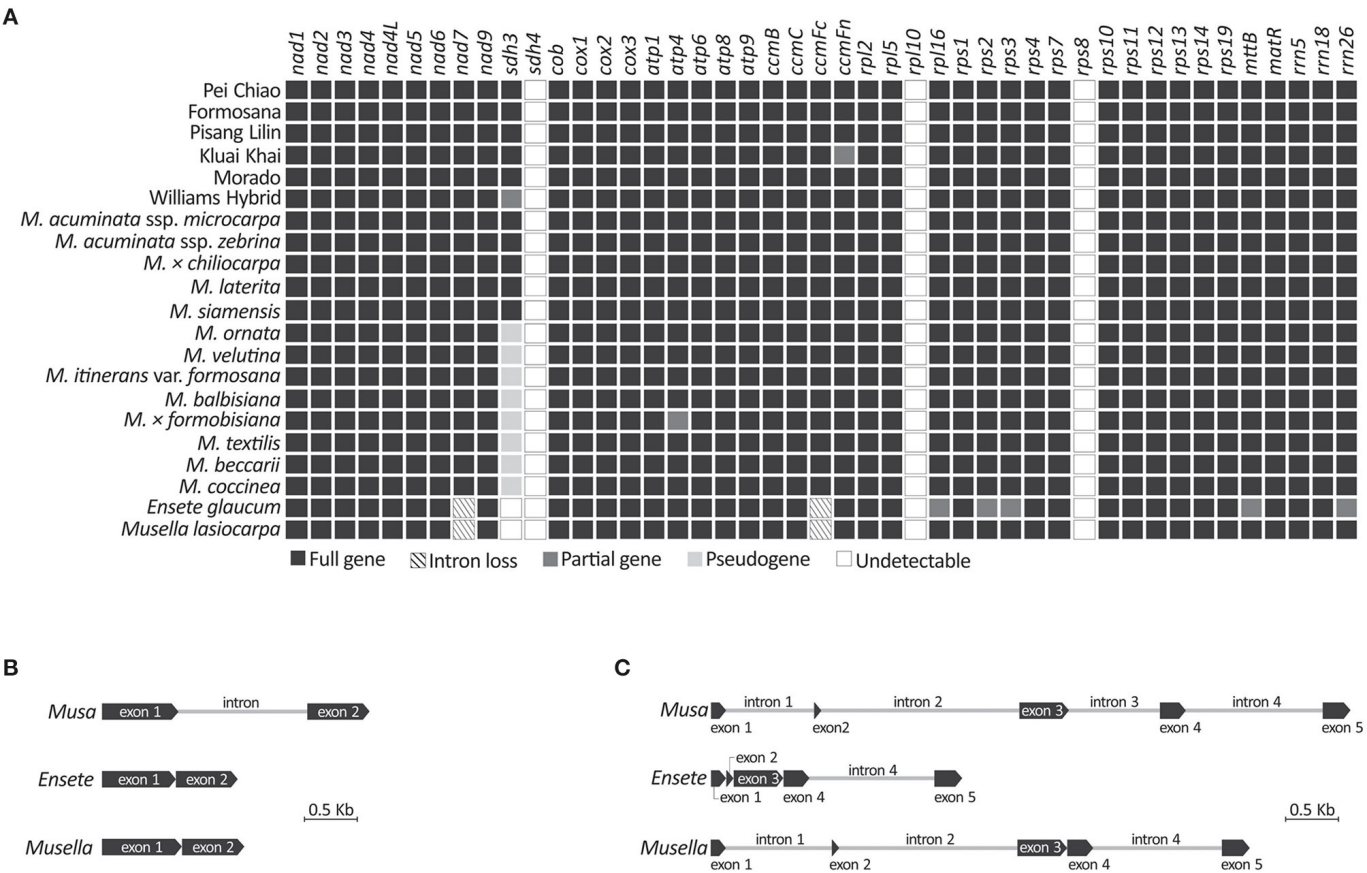
Comparisons of mitochondrial genes recovered from genome skimming. **(A)** Presence or absence of mitochondrial genes in the sampled banana genome assemblies. **(B)** Intron loss from *ccmFc*. **(C)** Intron loss from *nad7*.

We obtained 42 mitochondrial gene sequences, including 39 protein-coding and three rRNA genes (Figure 4A), from all sampled taxa. Three genes (i.e. *sdh4, rpl10*, and *rps8*) were not detected in our mitochondrial scaffolds though they are often present in angiosperm mitochondria (Chen et al., 2017). Although *sdh3* was previously detected in *Musa* based on Southern blot hybridizations (Adams et al., 2002), it was reported as either truncated at the 5'-region or premature due to early stop codons. Therefore, we treated *sdh3* as a pseudogene in all sampled *Musa* taxa, except for *M. acuminata, M*. × *chiliocarpa, M. laterita*, and *M. siamensis* whose *sdh3* remains functional (Supplementary Figure 3; Figure 4A). We did not detect *sdh3* in *Ensete* and *Musella*. They also lack the intron in *ccmFc* (Figure 4B). In addition, there are four *nad7* introns in *Musa*, but only one (intron 4) and three (introns 1, 2, and 4) are retained in *Ensete* and *Musella*, respectively (Figure 4C), suggesting that *Ensete* likely lost *nad7i1* and *nad7i2* after it diverged from *Musella*.

### Organellar Genome Phylogenies as a Useful Method to Study Banana Genealogy

**Figure 5:**
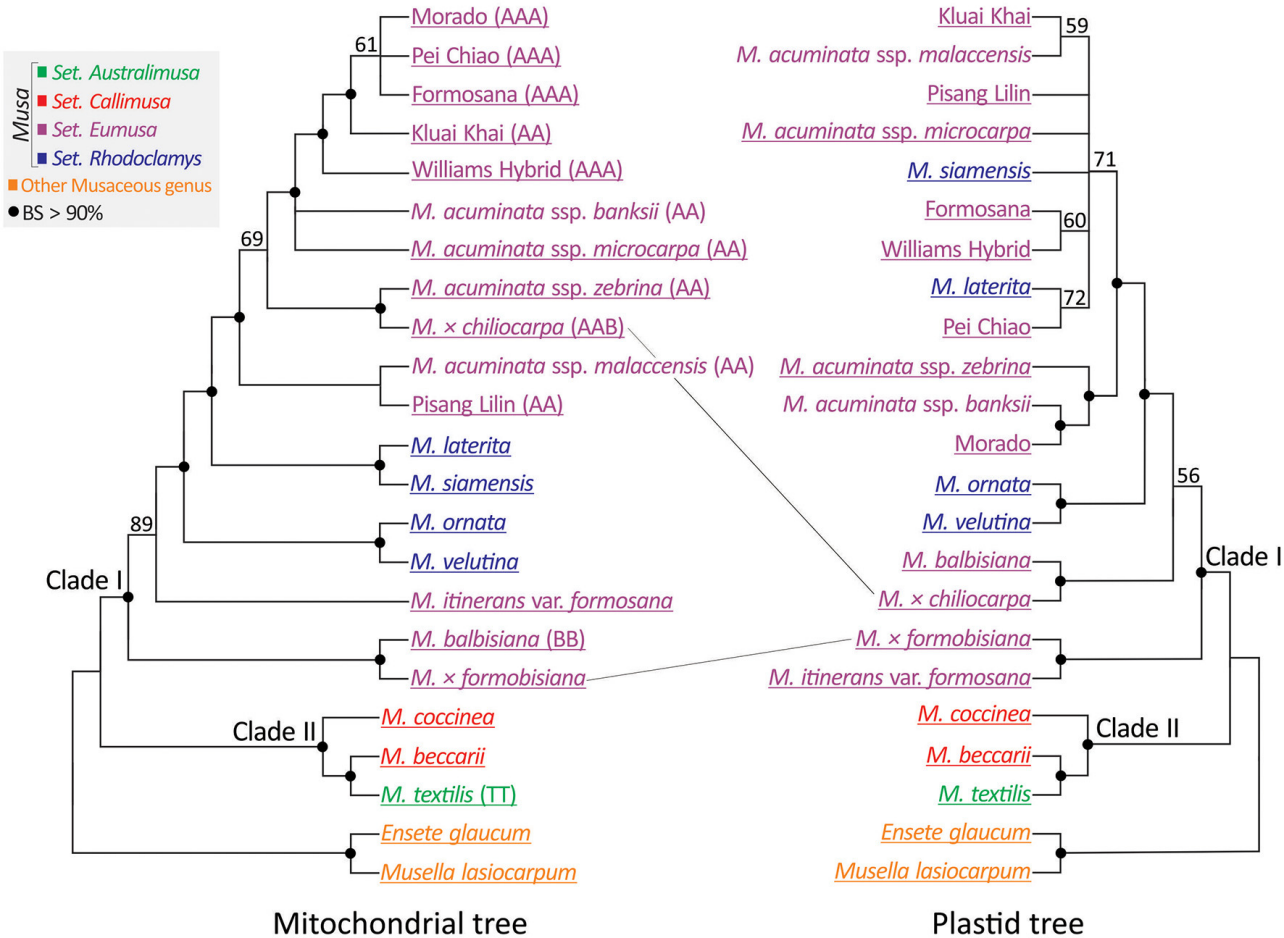
Comparing mitochondrial (left panel) and plastid (right panel) trees reveals different placement of two interspecific hybrid bananas. Genome types (if known) are indicated within parentheses. Taxa underlined are sequenced in this study. BS, bootstrap support.

A concatenated mitochondrial gene matrix was generated from the 21 newly sequenced accessions and two (*M. acuminata* ssp. *malaccensis* and *M. acuminata* ssp. *banksii*) from the Banana Genome Hub database (Supplementary Table 2). Subsequently, this matrix was used to construct an unrooted ML tree (Figure 5: left panel). To compare the mitochondrial to the plastid phylogeny, we reconstructed a plastid phylogenomic tree of a reduced set of taxa (Figure 5: right panel). Both trees support Clades I and II within *Musa* but differ in the placements of *M*. × *formobisiana* and *M*. × *chiliocarpa*. The former is placed as sister to *M. balbisiana* with a bootstrap value >90% in the mitochondrial tree, but as a sister of *M. itinerans* var. *formosana* in the plastid trees (Figures 2, 5). Since *M*. × *formobisiana* was a hybrid between *M. itinerans* var. *formosana* (maternal) and *M. balbisiana* (paternal; Chiu et al., 2017), this discrepancy implies heterogeneous transmission of cytoplasmic DNA, in line with the different modes of inheritance of these organelles (Fauré et al., 1994). In addition, *M*. × *chiliocarpa* is nested within the accessions of *M. acuminata* in the mitochondrial tree rather than a sister to *M. balbisiana* in the plastid gene trees (Figures 2, 5). Thus, we infer that *M*. × *chiliocarpa*'s AAB genotype is likely derived from a maternal parent *M. balbisiana* and a paternal parent *M. acuminata*. Collectively, our results demonstrate that comparisons of the two organellar phylogenies facilitate identification of bananas' parentage.

### Paternally Inherited Banana Mitogenomes Show Significantly Elevated Nucleotide Substitution Rates

**Figure 6:**
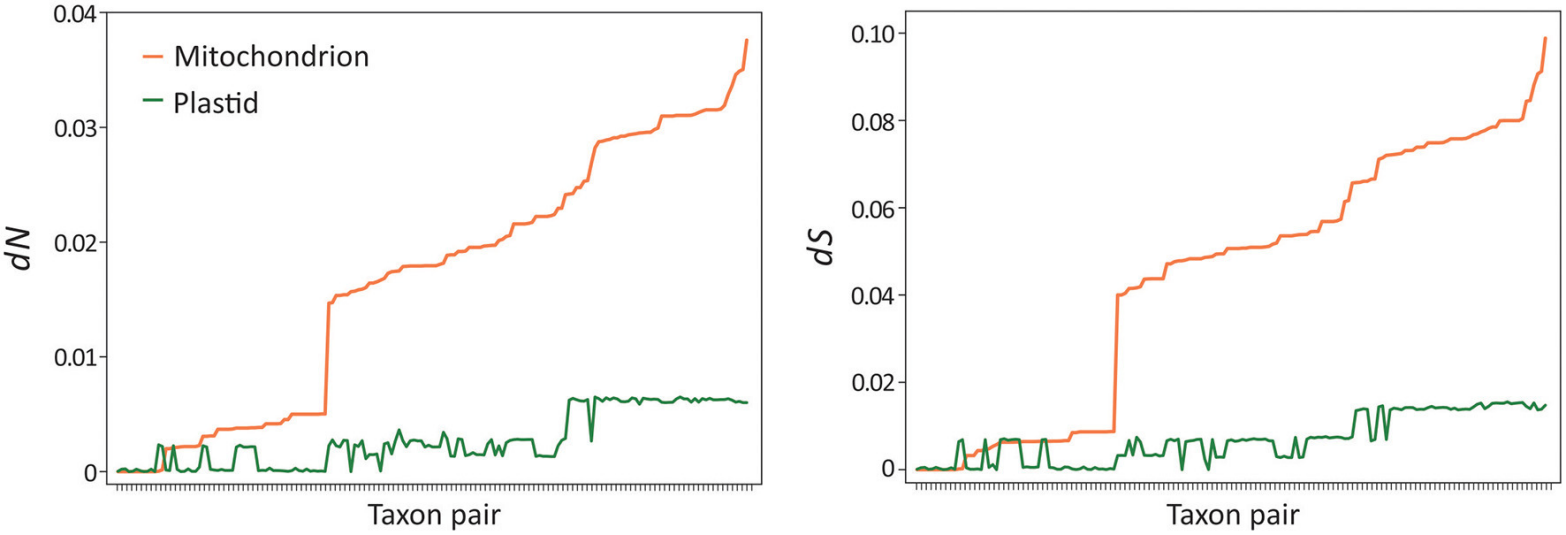
Elevated nucleotide substitution rates in banana mitogenomes. Paired *t*-tests indicate that both non-synonymous (*dN, P* = 1.19 × 10^−42^) and synonymous (*dS, P* = 2.77 × 10^−42^) rates are significantly elevated in mitochondria compared to plastids after the Bonferroni correction. Taxon pairs were sorted based on mitochondrial rates from the slowest to the fastest.

*Musa* mitogenomes are paternally inherited, unlike most seed plants where they are passed down from maternal parents (Greiner et al., 2015). This raises a question: did the paternal inheritance mode affect mitogenome evolution in *Musa*? To address this question, we estimated and compared synonymous (*dS*) and non-synonymous (*dN*) substitution rates between plastid and mitochondrial genes in *Musa*. We used pairwise rather than tree-based methods in estimating the substitution rates because of the differences in topology between the two organellar trees (Figure 5). Paired *t*-tests indicate that mitochondrial genes exhibit significantly higher nucleotide substitution rates than plastid loci at both *dN* (*P* = 1.19 × 10^−42^) and *dS* (*P* = 2.77 × 10^−42^) sites after the Bonferroni correction (Figure 6). Similarly, significantly elevated rates of nucleotide substitutions are also observed in mitochondrial rRNA genes compared to plastids (Supplementary Figure 4). These results contradict the previous suggestions that mitochondrial genes evolve slower than plastid loci (Wolfe et al., 1987; Drouin et al., 2008; Smith, 2015). Therefore, paternal inheritance likely plays an influential role on the evolution of *Musa* mitogenomes.

## Discussion

In this study, we employed genome skim data and recovered plastomes, 45S rDNAs, and mitochondrial gene sequences to re-assess the banana phylogeny and organelle inheritance modes. Numerous molecular studies have concluded that the three banana genera are monophyletic and *Musa* can be subdivided into two clades rather than four sections (see review in Häkkinen, 2013). Our results also support the two intrageneric clades in *Musa*, across trees inferred from plastid, 45S rDNA, or mitochondrial sequences. The plastid tree shown in Figure 2 reveals species-level paraphyly in *M. textilis, M. ornata*, and the accessions of *M. acuminata*. The former two are likely due to specimen misidentification as shown in our *matK* tree (Supplementary Figure 2). Taxonomic misidentification often leads to conflicting results because of difficulty in determining banana species, particularly when the specimen is incomplete (Li et al., 2010) or dried. However, taxonomic misidentification cannot account for the paraphyly of *M. acuminata* in the plastid tree as we found that this species is monophyletic in our mitochondrial tree. Previously, the paraphyly of *M. acuminata* and its cultivars was noted in several molecular studies based on the combination of a few plastid loci and ITS (Li et al., 2010; Janssens et al., 2016; Lamare et al., 2017) or low-copy nuclear genes (Christelová et al., 2011). Here we propose mitochondrial genes as reliable markers for banana phylogenetics because they eliminated species-level paraphyly from incomplete lineage sorting or genetic introgression (Funk and Omland, 2003; McKay and Zink, 2010). Particularly, rapid radiation was proposed to take place after the divergence of *M. acuminata* from *M. balbisiana* (Rouard et al., 2018).

Tracing the origins of hybrid bananas has previously heavily relied on restriction fragment length polymorphisms from several loci, including ITS, plastid, and mitochondrial sequences (Carreel et al., 2002; Nwakanma et al., 2003; Swangpol et al., 2007; Boonruangrod et al., 2008). We demonstrate that we can confidently infer two cultivated bananas' parentages from plastid and mitochondrial genes retrieved using the genome skimming method (Figure 5). Distinct transmission of these two organellar genomes allows identification of both paternal and maternal parents. *M*. × *chiliocarpa* is the second banana cultivar with the AAB genotype from a paternal *M. acuminata* and a maternal *M. balbisiana* after Pisang Rajah (Boonruangrod et al., 2008; De Langhe et al., 2010). *M* × *formosibiana* was also correctly inferred as the hybrid of a paternal parent *M. balbisiana* and a maternal parent *M. itinerans* var. *formosana* (Chiu et al., 2017).

Paternal inheritance of mitochondrial genomes is rare in seed plants. It has only been reported in some conifers (see review in Mogensen, 1996), bananas (Fauré et al., 1994; this study), cucumbers (Havey, 1997; Park et al., 2021), and melons (Zhao et al., 2014). These exceptional cases contradict the view that female-derived organelles are preferred as an evolutionary consequence of avoiding higher mutational loads in male gametes (Greiner et al., 2015). Indeed, the paternal organelles have significantly elevated rates of nucleotide substitutions in both conifers (Whittle and Johnston, 2002) and bananas (this study). Organellar genomes of sperm cells have low copy numbers and may exert stronger genetic bottleneck compared to egg cells, eventually leading to genetic drifts and elevated rates of nucleotide substitutions (Greiner et al., 2015). Additionally, low genome copy numbers also disrupt DNA repairs through recombination, resulting in accumulations of mismatches, as proposed in the *Silene conica* mitogenomes (Broz et al., 2021).

Yet, how these paternally inherited organelles deal with high mutational load and avoid mutational meltdown remain unknown. When and how did paternally-inherited mitochondria emerge in banana also requires further scrutiny. Shen et al. (2015) demonstrated that mitogenomes in the male generative cells of cucumbers are protected from DNA degradation by DNA-binding proteins, thus enabling paternally inherited mitochondria in cucumbers. Whether a similar mechanism also applies to male generative cells in banana species is worthy of investigation in future studies.

In conclusion, we show that 45S rDNA sequences and organellar genome sequences are informative for banana genealogy and phylogeny. As the cost of NGS continues to decrease, recovery of reliable genomic data from genome skimming will be affordable and routinely applicable for banana phylogenetic and genealogical studies. Additionally, our study supports the male-driven evolution in bananas: paternally inherited mitochondrial genes have higher substitution rates than maternally inherited plastid loci. This case further supports the idea that maternal inheritance evolved as the predominant organellar genome inheritance mode to escape higher mutational load in male gametes.

## Data Availability Statement

The original contributions presented in the study are publicly available. The DNA data generated in this study are deposited in GenBank with the accession numbers: LC603789–LC612134 and the SRA bioproject: PRJNA744736.

## Author Contributions

S-MC conceived and designed the study. C-SW performed the analyses. H-LC and C-PC prepared the banana specimens. C-SW, ES, and S-MC wrote the manuscript. All the authors checked and approved the final version.

## Conflict of Interest

The authors declare that the research was conducted in the absence of any commercial or financial relationships that could be construed as a potential conflict of interest.

## Publisher's Note

All claims expressed in this article are solely those of the authors and do not necessarily represent those of their affiliated organizations, or those of the publisher, the editors and the reviewers. Any product that may be evaluated in this article, or claim that may be made by its manufacturer, is not guaranteed or endorsed by the publisher.
