# Corrigendum: Reassessing Banana Phylogeny and Organelle Inheritance Modes Using Genome Skimming Data

**DOI:** 10.3389/fpls.2021.771777

**Published:** 2021-10-01

**Authors:** Chung-Shien Wu, Edi Sudianto, Hui-Lung Chiu, Chih-Ping Chao, Shu-Miaw Chaw

**Affiliations:** ^1^Biodiversity Research Center, Academia Sinica, Taipei, Taiwan; ^2^Plant Germplasm Division, Taiwan Agricultural Research Institute, Taichung, Taiwan; ^3^Taiwan Banana Research Institute, Pingtung, Taiwan

**Keywords:** banana, genome skimming, cytoplasmic inheritance, nrDNA, mitogenome, plastome

In the original article, in the section Mitochondrial Gene Repertoire of Musaceae, the sentence “They also lack the intron in *ccmFn* (**Figure 4B**).” should have read “They also lack the intron in *ccmFc* (**Figure 4B**).”

In the associated caption to Figure 4B, “(B) Intron loss from *ccmFn*.” should have read “(B) Intron loss from *ccmFc*.”

The authors apologize for this error and state that this does not change the scientific conclusions of the article in any way. The original article has been updated.

## Publisher's Note

All claims expressed in this article are solely those of the authors and do not necessarily represent those of their affiliated organizations, or those of the publisher, the editors and the reviewers. Any product that may be evaluated in this article, or claim that may be made by its manufacturer, is not guaranteed or endorsed by the publisher.

