# Supplementary material for: Reassessing Banana Phylogeny and Organelle Inheritance Modes Using Genome Skimming Data
Source: Front Plant Sci. 2021 Aug 11;12:713216. doi: 10.3389/fpls.2021.713216 (PMC8385209; doi:10.3389/fpls.2021.713216)
Supplement: Supplementary file 1 [file Data_Sheet_1.PDF]

*Supplementary Material*

# Reassessing banana phylogeny and organelle inheritance modes using genome skim data

**Chung-Shien Wu[1†], Edi Sudianto[1†], Hui-Lung Chiu[2], Chih-Ping Chao[3], Shu-Miaw Chaw[1*]**

[1]Biodiversity Research Center, Academia Sinica, Taipei 11529, Taiwan
[2]Taiwan Agricultural Research Institute, Taichung 413, Taiwan
[3]Taiwan Banana Research Institute, Pingtung 90442, Taiwan
[†]These authors contributed equally to this work
*** Correspondence:**
Corresponding Author
smchaw@sinica.edu.tw

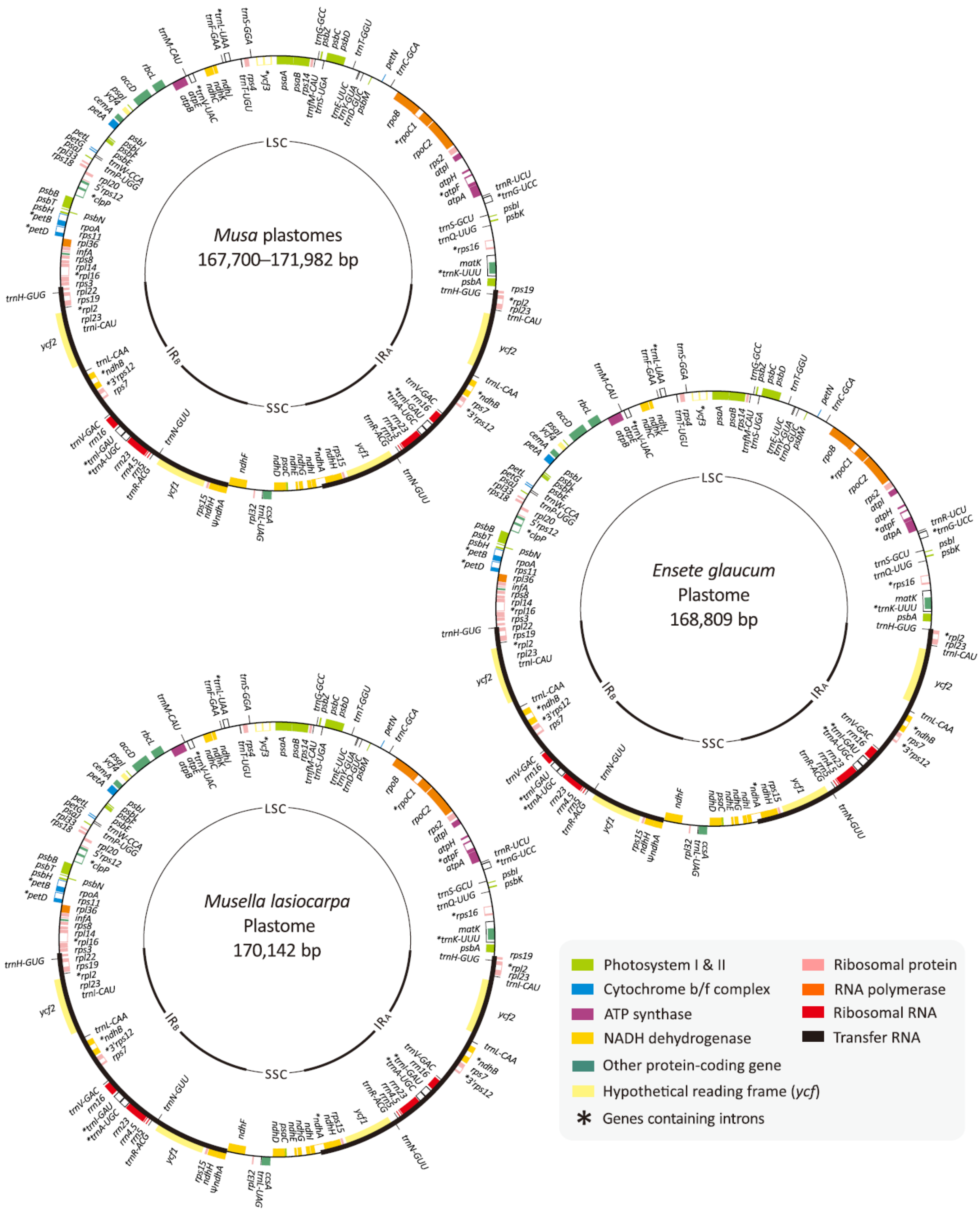


**Supplementary Figure S1.** *Musa*, *Ensete*, and *Musella* plastome maps. Genes inside and outside the outermost circle are transcribed clockwise and counterclockwise, respectively. IR, inverted repeat; LSC, large single-copy region; SSC, small single-copy region.

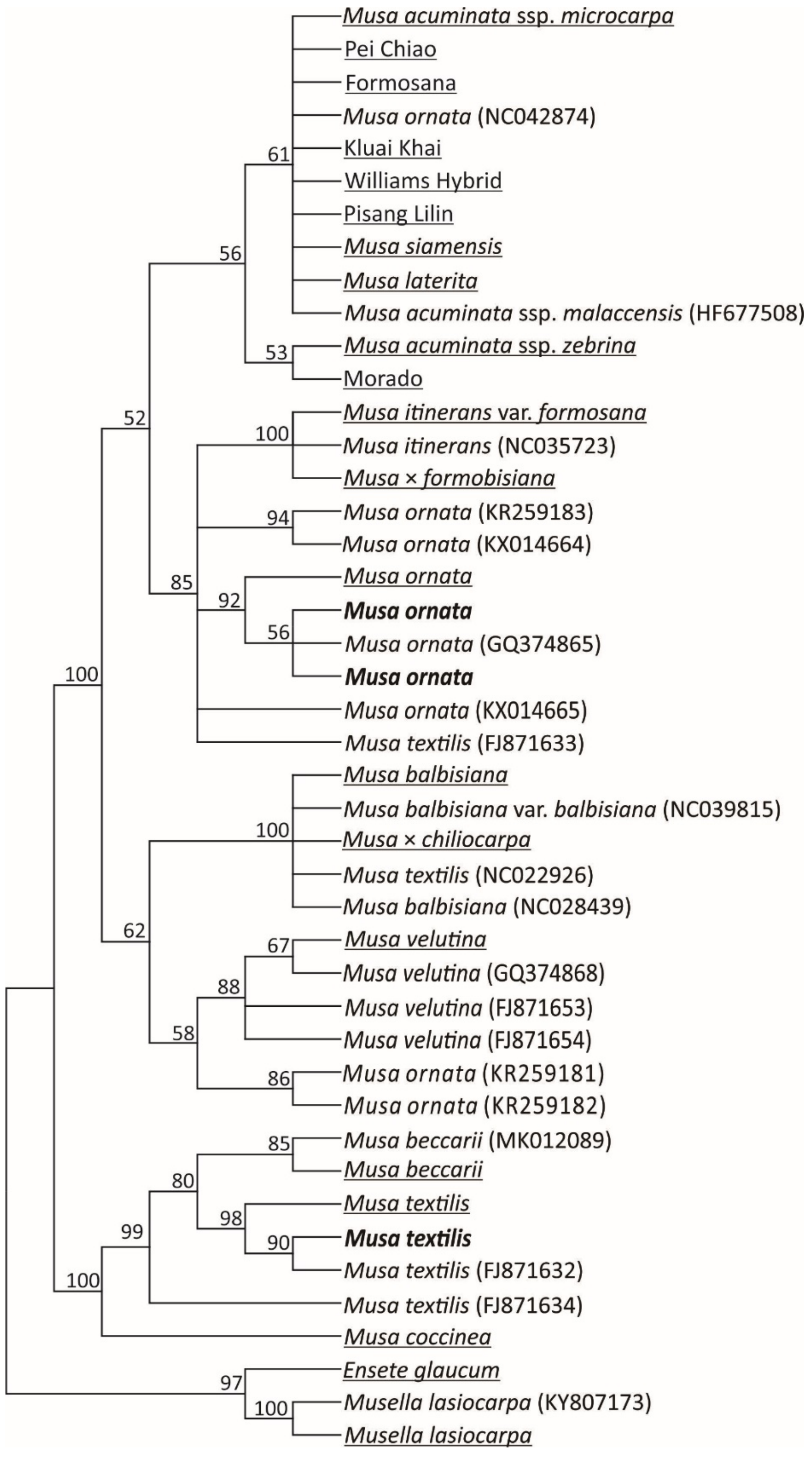


**Supplementary Figure S2.** An ML tree inferred from *matK* sequences. The underlined and bolded taxa are accessions whose plastomes and *matK* genes are sequenced in this study. Publicly available data retrieved from GenBank are labeled with their accession numbers within parentheses. Values along branches are bootstrap supports estimated from 1,000 replicates.

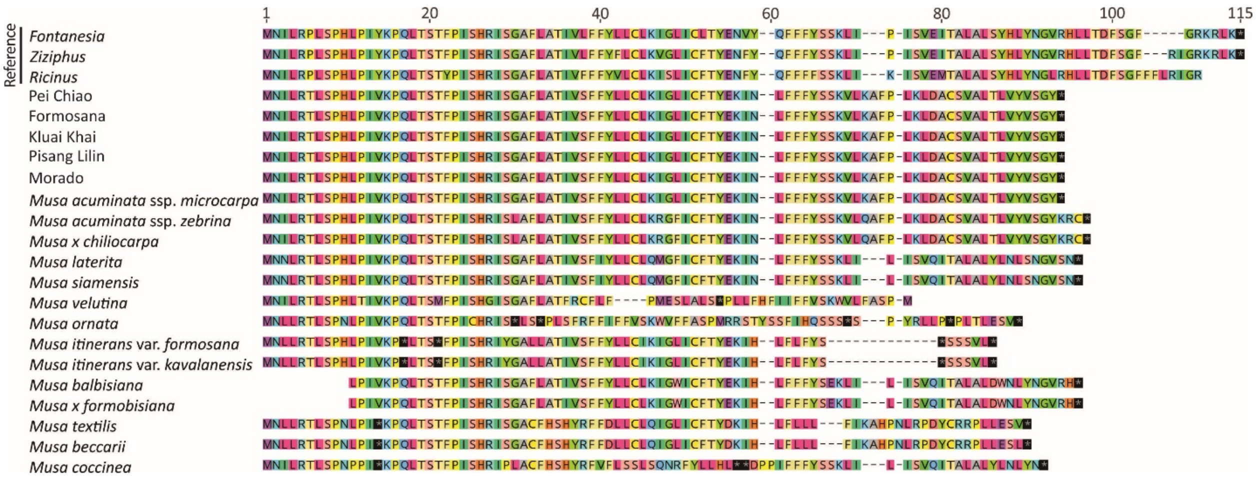


**Supplementary Figure S3.** An amino acid alignment of mitochondrial *sdh3* genes.

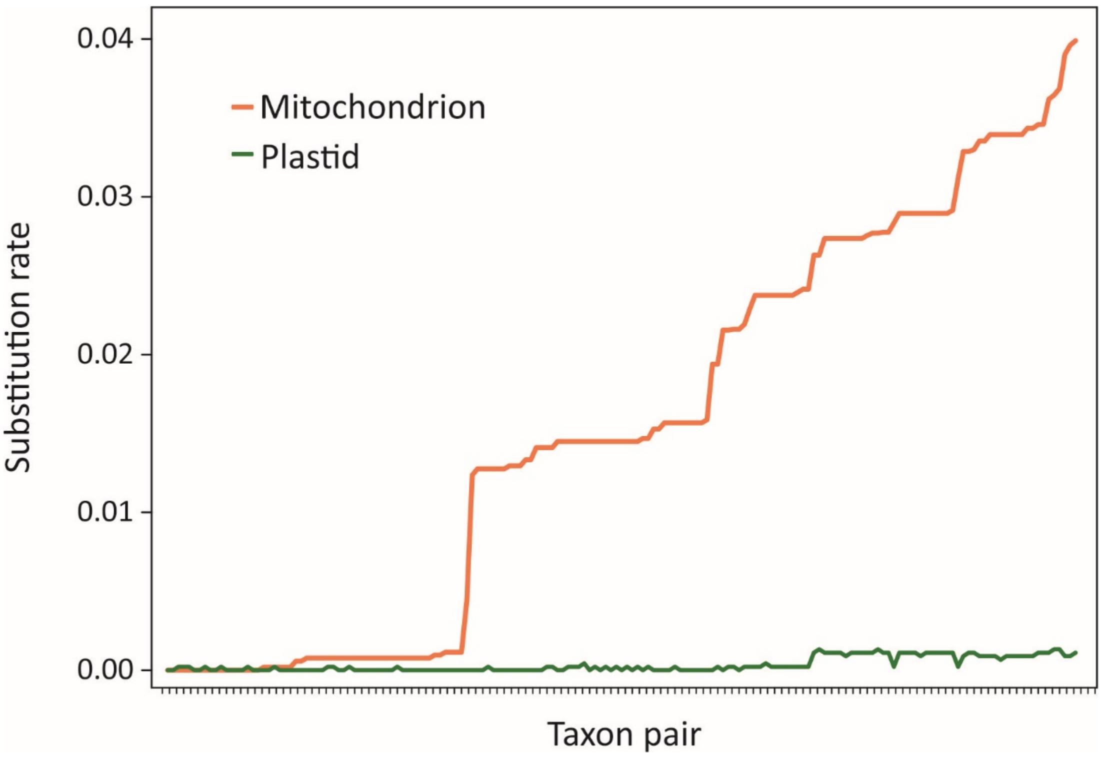


**Supplementary Figure S4.** Comparing nucleotide substitution rates of organellar rRNAs in bananas. A paired t-test indicates significantly elevated mitochondrial nucleotide substitution rate compared to plastids after the Bonferroni correction ($P = 2.21 \times 10^{-34}$). Taxon pairs were sorted based on the mitochondrial rate from the slowest to the fastest.

**Supplementary Table S1.** Summary of the sequencing amount and targeting scaffolds for the 21 sequenced bananas

| Species/common name | No. of reads (million) | No. of bases (Gb) | Targeting scaffold | | |
|---|---|---|---|---|---|
| | | | 45S rDNA | Plastid | Mitochondrion |
| Pei Chiao | 14.45 | 2.18 | 1/6.8[1] | 1/170.3 | 29/1,380.4 |
| Formosana | 13.89 | 2.09 | 1/6.8 | 1/170.3 | 22/1,598.8 |
| Kluai Khai | 14.14 | 2.13 | 1/6.8 | 1/170.0 | 28/1,367.2 |
| Morado | 14.52 | 2.19 | 1/6.8 | 1/169.7 | 27/1,197.6 |
| Pisang Lilin | 14.99 | 2.25 | 1/6.8 | 1/170.6 | 28/1,842.5 |
| Williams Hybrid | 14.54 | 2.19 | 1/6.8 | 1/169.9 | 25/1,404.8 |
| *Musa acuminata* ssp. *zebrina* | 14.71 | 2.21 | 1/6.8 | 1/169.9 | 29/1,845.8 |
| *Musa acuminata* ssp. *microcarpa* | 13.86 | 2.06 | 1/6.8 | 1/170.5 | 25/1,890.7 |
| *Musa balbisiana* | 14.59 | 2.19 | 1/6.8 | 1/169.9 | 17/2,244.7 |
| *Musa itinerans* var. *formosana* | 14.54 | 2.19 | 1/6.8 | 1/171.5 | 26/2,545.7 |
| *Musa × chiliocarpa* | 13.46 | 2.03 | 1/6.8 | 1/171.0 | 22/1,722.6 |
| *Musa × formobisiana* | 14.66 | 2.01 | 1/6.8 | 1/172.0 | 24/1,977.8 |
| *Musa beccarii* | 13.77 | 2.07 | 1/6.5 | 1/168.2 | 26/694.8 |
| *Musa coccinea* | 15.07 | 2.27 | 1/6.8 | 1/167.7 | 25/2,115.4 |
| *Musa laterita* | 14.11 | 2.13 | 1/6.8 | 1/170.1 | 26/2,369.5 |
| *Musa ornata* | 15.67 | 2.34 | 1/6.8 | 1/170.3 | 26/3,104.4 |
| *Musa velutina* | 14.35 | 2.16 | 1/6.8 | 1/170.1 | 26/1,458.6 |
| *Musa siamensis* | 12.71 | 1.87 | 1/6.8 | 1/170.3 | 27/2,243.4 |
| *Musa textilis* | 14.46 | 2.14 | 1/6.8 | 1/168.2 | 23/1,633.3 |
| *Ensete glaucum* | 15.42 | 2.31 | 1/6.8 | 1/168.8 | 32/514.1 |
| *Musella lasiocarpa* | 15.39 | 2.30 | 1/6.8 | 1/170.1 | 21/2,340.2 |

[1]No. of scaffolds/total length of scaffolds (Kb)

**Supplementary Table S2**. Analyzed sequences and their GenBank accession numbers in this study

| Species/common name | Collection | GenBank accession number | | | SRA accession number |
|---|---|---|---|---|---|
| | | 45S rDNA | Plastome | Mitochondrial gene | |
| Pei Chiao | This study | LC610764 | LC609626 | LC610986–LC611027 | SRX11421497 |
| Formosana | This study | LC610757 | LC603789 | LC611028–LC611069 | SRX11421498 |
| Kluai Khai | This study | LC610760 | LC609620 | LC612017–LC612057 | SRX11421502 |
| Morado | This study | LC610762 | LC609770 | LC611112–LC611153 | SRX11421499 |
| Pisang Lilin | This study | LC610765 | LC609627 | LC611070–LC611111 | SRX11421500 |
| Williams Hybrid | This study | LC610769 | LC609628 | LC612058–LC612098 | SRX11421496 |
| *Musa acuminata* ssp. *banksii* | Publicly available | n.a. | MK210631 | Banana Genome Hub (Genome assembly v2) | n.a. |
| *Musa acuminata* ssp. *malaccensis* | Publicly available | n.a. | HF677508 | Banana Genome Hub (Genome assembly v2) | n.a. |
| *Musa acuminata* ssp. *zebrina* | This study | LC610751 | LC609622 | LC610944–LC610985 | SRX11421501 |
| *Musa acuminata* ssp. *microcarpa* | This study | LC610750 | LC609621 | LC610902–LC610943 | SRX11421490 |
| *Musa balbisiana* | Publicly available | n.a. | NC028439 | n.a. | n.a. |
| *Musa balbisiana var. balbisiana* | Publicly available | n.a. | NC039815 | n.a. | n.a. |
| *Musa balbisiana* | This study | LC610752 | LC609623 | LC611772–LC611812 | SRX11421503 |
| *Musa itinerans* | Publicly available | n.a. | NC035723 | n.a. | n.a. |
| *Musa itinerans* var. *formosana* | This study | LC610758 | LC609773 | LC611362–LC611402 | SRX11421508 |
| *Musa* × *chiliocarpa* | This study | LC610754 | LC609625 | LC611154–LC611195 | SRX11421505 |
| *Musa* × *formobisiana* | This study | LC610756 | LC609772 | LC611813–LC611852 | SRX11421507 |
| *Musa beccarii* | Publicly available | n.a. | MK012089 | n.a. | n.a. |
| *Musa beccarii* | This study | LC610753 | LC609624 | LC611894–LC611934 | SRX11421504 |
| *Musa coccinea* | This study | LC610755 | LC609771 | LC611935–LC611975 | SRX11421506 |
| *Musa laterita* | This study | LC610761 | LC609775 | LC611196–LC611237 | SRX11421509 |
| *Musa ornata* | This study | LC610763 | LC609776 | LC611280–LC611320 | SRX11421491 |
| *Musa ornata* | Publicly available | n.a. | NC042874 | n.a. | n.a. |
| *Musa velutina* | This study | LC610768 | LC609778 | LC611321–LC611361 | SRX11421494 |
| *Musa siamensis* | This study | LC610766 | LC610771 | LC611238–LC611279 | SRX11421492 |
| *Musa textilis* | Publicly available | n.a. | NC022926 | n.a. | n.a. |
| *Musa textilis* | This study | LC610767 | LC609777 | LC611853–LC611893 | SRX11421493 |
| *Ensete glaucum* | This study | LC610749 | LC610748 | LC612099–LC612134 | SRX11421489 |
| *Musella lasiocarpa* | Publicly available | n.a. | KY807173 | n.a. | n.a. |
| *Musella lasiocarpa* | This study | LC610770 | LC610747 | LC611976–LC612016 | SRX11421495 |
| *Heliconia collinsiana* | Publicly available | n.a. | JX088660 | n.a. | n.a. |
| *Ravenala madagascariensis* | Publicly available | n.a. | NC022927 | n.a. | n.a. |

n.a.: Not available
